# Integrative Meta-Assembly Pipeline (IMAP): Chromosome-level genome assembler combining multiple *de novo* assemblies

**DOI:** 10.1371/journal.pone.0221858

**Published:** 2019-08-27

**Authors:** Giltae Song, Jongin Lee, Juyeon Kim, Seokwoo Kang, Hoyong Lee, Daehong Kwon, Daehwan Lee, Gregory I. Lang, J. Michael Cherry, Jaebum Kim

**Affiliations:** 1 School of Computer Science and Engineering, Pusan National University, Busan, South Korea; 2 Department of Biomedical Science and Engineering, Konkuk University, Seoul, South Korea; 3 Department of Biological Sciences, Lehigh University, Bethlehem, PA, United States of America; 4 Department of Genetics, Stanford University School of Medicine, Stanford, California, United States of America; Saint Louis University, UNITED STATES

## Abstract

**Background:**

Genomic data have become major resources to understand complex mechanisms at fine-scale temporal and spatial resolution in functional and evolutionary genetic studies, including human diseases, such as cancers. Recently, a large number of whole genomes of evolving populations of yeast (*Saccharomyces cerevisiae* W303 strain) were sequenced in a time-dependent manner to identify temporal evolutionary patterns. For this type of study, a chromosome-level sequence assembly of the strain or population at time zero is required to compare with the genomes derived later. However, there is no fully automated computational approach in experimental evolution studies to establish the chromosome-level genome assembly using unique features of sequencing data.

**Methods and results:**

In this study, we developed a new software pipeline, the integrative meta-assembly pipeline (IMAP), to build chromosome-level genome sequence assemblies by generating and combining multiple initial assemblies using three *de novo* assemblers from short-read sequencing data. We significantly improved the continuity and accuracy of the genome assembly using a large collection of sequencing data and hybrid assembly approaches. We validated our pipeline by generating chromosome-level assemblies of yeast strains W303 and SK1, and compared our results with assemblies built using long-read sequencing and various assembly evaluation metrics. We also constructed chromosome-level sequence assemblies of *S*. *cerevisiae* strain Sigma1278b, and three commonly used fungal strains: *Aspergillus nidulans* A713, *Neurospora crassa* 73, and *Thielavia terrestris* CBS 492.74, for which long-read sequencing data are not yet available. Finally, we examined the effect of IMAP parameters, such as reference and resolution, on the quality of the final assembly of the yeast strains W303 and SK1.

**Conclusions:**

We developed a cost-effective pipeline to generate chromosome-level sequence assemblies using only short-read sequencing data. Our pipeline combines the strengths of reference-guided and meta-assembly approaches. Our pipeline is available online at http://github.com/jkimlab/IMAP including a Docker image, as well as a Perl script, to help users install the IMAP package, including several prerequisite programs. Users can use IMAP to easily build the chromosome-level assembly for the genome of their interest.

## Introduction

As sequencing technology is advancing in terms of cost efficiency and sequencing accuracy, whole genome sequencing is being used more frequently in modern genetics. This sequencing approach provides innovative temporal and spatial resolution to examine fundamental problems in population and evolutionary genomics [1], and empowers the study of human genetic disease, such as studies of cancer [2]. Recently, several laboratories have performed short-term evolution studies in evolving populations of *Saccharomyces cerevisiae* using whole genome sequence data to understand evolutionary patterns associated with cell population growth [3, 4].

Despite the substantial contributions that whole genome sequencing approaches have made to experimental evolutionary studies, some sequence variations might escape detection if they are examined based on the mapping of short-reads to a diverged reference genome. For example, the yeast strain W303, used in many evolution experiments, differs from the reference S288C strain at approximately 9,500 nucleotide positions [5]. If structural or copy number variations occur in strain-specific genomic regions that are not present in the S288C reference strain, the lack of a true chromosome-level genome assembly of a specific strain is especially problematic.

Building a *de novo* sequence assembly for the target genome permits genome analyses of novel sequences beyond the reference. However, *de novo* assembly is a computationally challenging problem because of the short length of the read sequences that must be merged into a complete chromosome-level sequence of the target genome. Instead of a complete continuous chromosome sequence, short-reads are built into fragmented chunks of continuous nucleotides, termed contigs, and into supercontigs, termed scaffolds, especially if frequent repeat elements are present in the genome [6]. Long-read sequencing from third generation sequencing technologies, such as Pacific Biosciences (PacBio) and Oxford Nanopore, can greatly improve the quality of sequence assembly; however, they are currently expensive, and are more error-prone than short-read sequencing [7, 8]. Instead of using these expensive technologies, we designed a new computational pipeline to resolve this issue using only cost-effective short-read sequencing data, which we term the integrative meta-assembly pipeline (IMAP).

One approach to improving the continuity of the assembly is to combine multiple versions of sequence assemblies into larger scaffolds for a given target genome (for examples, see references [9, 10]). However, this approach cannot produce a chromosome-level genome assembly. Another strategy is to use a reference-guided approach that uses a set of existing genome assemblies that are closely related to the target sequence to fill assembly gaps, and sort the order of contigs or scaffolds [11−13]. Unfortunately, this approach cannot address the issue that different assembly approaches may yield different assembly outputs.

To overcome these limitations, we developed a new hybrid assembly approach that combines two recently published tools, RACA [13] and DESCHRAMBLER [14], to order scaffolds and build a chromosome-level assembly. RACA and DESCHRAMBLER are used to increase the contiguity of initial assemblies, and to generate a meta-assembly that integrates multiple initial assemblies, respectively. We also iteratively refined the assembly to improve its accuracy at the nucleotide level. We integrated this method into a pipeline to serve other groups in the fields of population and evolutionary genomics, especially for studies of evolving populations using unicellular eukaryotic organisms. We built sequence assemblies of *S*. *cerevisiae* strains, W303, and SK1 automatically, and compared them with assemblies generated using long-read sequencing for validation [5, 15]. We demonstrated the utility of our pipeline by constructing a chromosome-level assembly for *S*. *cerevisiae* Sigma 1278b, a strain widely used as a model for pseudohyphal growth [16]. In addition to *S*. *cerevisiae*, we also applied IMAP for other fungal species such as *Aspergillus nidulans*, *Neurospora crassa*, and *Thielavia terrestris* that are used as model organisms in cell biology and genetics studies [17–19].

Our generalizable approach could establish a chromosome-level sequence assembly for the genome of a given strain, which maximizes the unique characteristics of short-read sequencing data from evolving populations based on *de novo* assembly, and covers strain-specific genomic regions. In studies where long-read sequencing is not available, such as those investigating short-term experimental evolution, our pipeline will be a useful tool to elucidate evolutionary dynamics with fine-scale temporal resolution.

## Materials and methods

### Data preparation and initial assembly

We obtained 40 datasets representing the initial time point of evolution studies using *Saccharomyces cerevisiae* W303 strain (NCBI Bioproject accession: PRJNA205542, [4]). Each set comprised Illumina paired-end short-read sequencing data at approximately 100× fold coverage (read length: 100 bases). After the 40 datasets were merged, the depth of the W303 sequencing data was approximately 4,000× coverage. For strain SK1, we used two sequencing datasets: One with approximately 474× coverage (NCBI Biosample accession: SAMN05792036, [20]), and the other with approximately 56× coverage (available at ftp://ftp.sanger.ac.uk/pub/users/dmc/yeast/SGRP2/input/strains/17A_Sc_SK1/, [21]). For yeast strain Sigma1278b, we used a data set with approximately 191× coverage (Biosample accession: SAMN03020229, [22]). The information of data for three fungal strains is available in S1 Table.

First, we filtered out sequencing reads based on ambiguous bases (Ns) and low-quality reads from the merged dataset. We then applied several *de novo* assembly methods using the collected short-read data. We compared their assembly results using major quality metrics that were used in the assembly evaluation consortium project called Assemblathon [23]. From this validation, we decided to use three *de novo* assembly tools: MaSurCA, SPAdes, and SOAPdenovo2 [24−26], using default parameters. If users wish to include other initial assemblies, they can easily add them at this step.

### Meta-assembly approach for chromosome level assembly

We extended the scaffolds of the three initial assemblies to a chromosome-level assembly using RACA [13] and DESCHRAMBLER [14]. In our pipeline, the contiguity of the initial assemblies was increased by RACA, and the meta-assembly from the improved initial assemblies was generated by DESCHRAMBLER (details of command lines and options are available at our GitHub website: http://github.com/jkimlab/IMAP). RACA requires a reference sequence that is close to the user’s target genome, and an outgroup with a known phylogeny. The order of scaffolds in each initial assembly was determined under the guidance of the reference sequence and the sequence of the outgroup, and the scaffolds were then enlarged to chromosome-level scaffolds using RACA. For *S*. *cerevisiae* strain genomes, we used the reference sequence from strain S288C, which was obtained from the *Saccharomyces* Genome Database (http://www.yeastgenome.org). For the outgroup in RACA, we used the genome sequence of *Naumovozyma dairenensis* (NCBI RefSeq: GCF_000227115.2). All RACA assembly outcomes in chromosome-level scaffolds were combined together into a consensus sequence using DESCHRAMBLER with the same reference sequence. DESCHRAMBLER was originally developed to reconstruct the ancestral genome, given the genome assemblies of multiple descendants. In this study, we applied DESCHRAMBLER to construct a meta-assembly by treating multiple initial assemblies as descendant assemblies and the meta-assembly as their common ancestor.

We then iteratively refined this chromosome-level assembly using Pilon (with default options in version 1.13; [27]), and GATK (with default options in version 3.7; [28]), using the original raw reads as input, and our assembly as the reference. We expected that a more accurate assembly is likely to have fewer SNPs, when SNPs are called based on the mapping of raw reads data against its own assembly, itself created using the raw reads. After mapping read sequences against an assembly sequence, SNPs were called using GATK. GATK is a pipeline for calling single nucleotide polymorphisms (SNPs). The nucleotides at SNP positions were corrected to the majority base present among the mapped nucleotides. If the majority base at a position was identical to the base in the assembly, the base at that position was unchanged. If the two mapped nucleotides at a position were tied in number, we also left the base at that position unchanged.

### Assembly validation

We used several evaluation metrics to assess the quality of the assembly. The metrics included basic assembly statistics, such as N50 (the shortest sequence length at 50% of the genome), MIN (the shortest scaffold), MAX (the longest scaffold), and total length. We also evaluated the assembly results using Bowtie2 [29] and GATK [28]. We calculated the portion of read pairs that were properly mapped out of mapped reads for paired-end sequencing data relative to our assembly sequence using Bowtie2. In addition, we counted the number of false positive SNPs called by GATK in the raw read data relative to its own assembly. If the new assembly is built more correctly, we would expect fewer SNPs than those observed in the original assembly.

In addition, for certain target genomes, such as yeast strains W303 and SK1, another version of their assembly was built by different research groups using long-read sequencing technology, such as PacBio (e.g. [5,15]). We compared our assembly results with those assemblies for validation.

We also used AGAPE (with default options; [22]) for evaluation. AGAPE is a pipeline to annotate chromosomal features in a given sequence. We measured the assembly accuracy based on the number of yeast genes correctly annotated using AGAPE for a given sequence assembly. We assumed that a sequence of higher quality would have more genes annotated accurately by AGAPE. We also investigated paralogous genes using the AGAPE gene annotation results, and compared them between our assembly and the PacBio assembly. Yeast paralogous genes were determined using BLAST [30] against the yeast reference genome. A total of 480 paralogous genes were grouped into 197 sets. The genome sequence segments including these paralogous genes were often missed in *de novo* assembly, because of their repetitiveness. Checking the annotation status for the paralogous genes was another way to assess assembly accuracy.

Synteny-level similarity between our IMAP assembly and the PacBio output was checked using LASTZ alignment (with version 1.02, with options–E = 150 –H = 2000 –K = 4300 –L = 2200 –M = 254 –O = 600 –T = 2 –Y = 15000; [31]). We mapped syntenic blocks that represented conserved genomic segments between two assembly sequences. We visualized the synteny mapping results using SynCircos, implemented in mySyntenyPortal [32]. We used this to verify the agreement of the order and orientation of large-scale genomic segments between the two assemblies.

## Results and discussion

### Overview of the Integrative Meta-Assembly Pipeline (IMAP)

**Fig 1:**
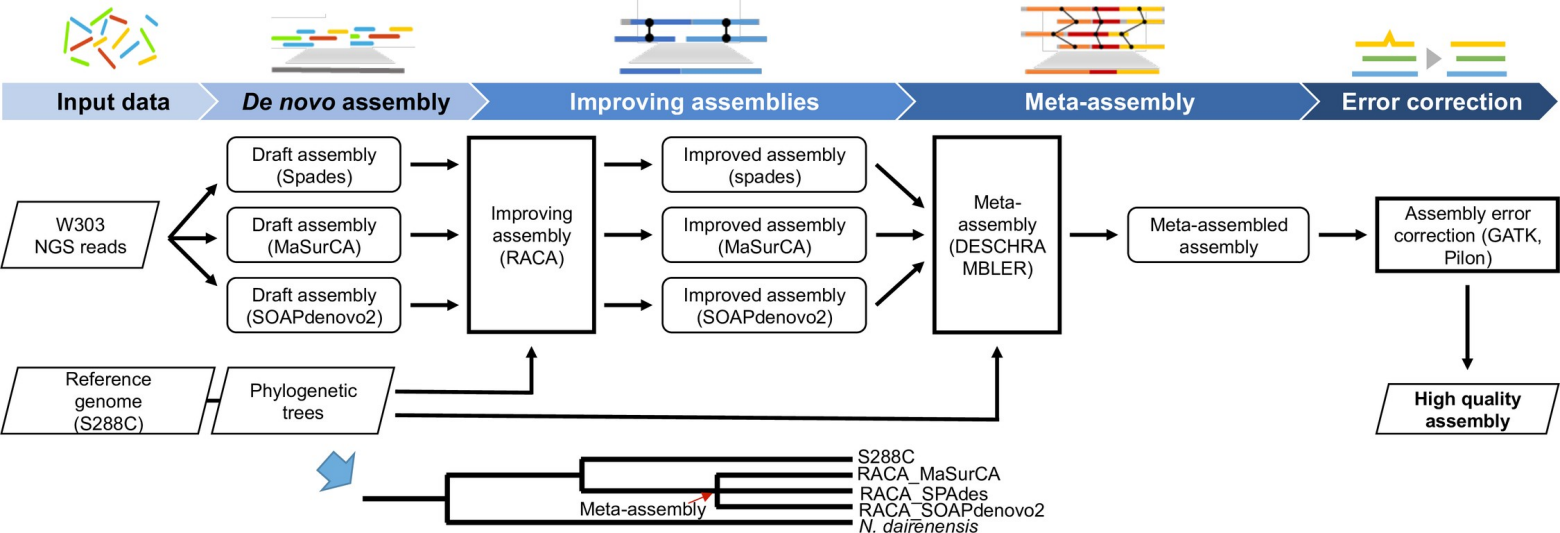
Data flow chart of the integrative meta-assembly pipeline (IMAP). Short raw sequencing data reads for a target genome were extended to initial scaffolds using *de novo* assembly software. We applied several *de novo* assembly tools, and produced several draft assemblies for the target genome. The order of scaffolds in each assembly was determined under the guidance of the reference sequence and the sequence of an outgroup; and the scaffolds were then enlarged to chromosome-level scaffolds using RACA. All RACA assembly outcomes in chromosome-level scaffolds were combined together into a consensus sequence, using DESCHRAMBLER. We then refined this consensus sequence at the nucleotide level using Pilon and GATK, and finished it as a chromosome-level sequence for the target genome. The tree in the bottom of this figure illustrates the phylogeny among the target genome, the reference, and the outgroup sequence. For the target sequence, the consensus of the three RACA assembly results obtained using the three *de novo* assembly tools (MaSurCA, SPAdes, and SOAPdenovo2) was obtained using DESCHRAMBLER.

We developed a software pipeline called IMAP to establish a chromosome-level sequence assembly of a user’s target genome given a collection of sequencing raw reads (see Fig 1). First, we generated initial draft assemblies using *de novo* assembly tools. Note that although we built the three initial draft assemblies based on MaSurCA, SPAdes, and SOAPdenovo2, respectively, more tools could be applied. These initial assemblies were combined and extended to larger scaffolds at the chromosome level using RACA and DESCHRAMBLER. The improved assembly was refined using Pilon and GATK error correction. All of these steps were implemented and integrated into one pipeline for automatic construction of the chromosome-level *de novo* assembly. Our pipeline is available on GitHub for users who wish to perform chromosome-level assembly using short-read sequencing data of their genome. This pipeline is ideally suited for laboratories that need to generate a strain specific chromosome-level genome assembly in a cost-effective manner. Once users input their own initial raw sequencing data (in the FASTQ format), the final assembly at the chromosome level can be automatically built by IMAP using a single command.

### Evaluation of chromosome-level assembly built by IMAP

We applied IMAP to the *S*. *cerevisiae* strain W303, one of the most commonly used strains in yeast studies [33]. We relied on W303 short-read sequence data from an experimental evolution study [4] that is available at NCBI SRA with Bioproject ID PRJNA205542. To construct the W303 sequence assembly, we used only samples from time point zero. We downloaded all 40 W303 datasets (each set has approximately 100× coverage) at the initial time point, and merged them to approximately 4,000× coverage data.

We generated three initial draft assemblies for this data using MaSurCA, SPAdes, and SOAPdenovo2. The chromosome-level assembly of the W303 strain genome was constructed using IMAP. We evaluated each step of our pipeline. To validate which step influenced the quality of the final assembly, we measured basic statistics for the outcome of each step in the pipeline, using metrics such as the length of the shortest contig (MIN), the length of the longest contig (MAX), and N50 of the assembly (see Table 1). According to the basic statistics of the assemblies, extending all initial draft assemblies to chromosome-level scaffolds and combining them into a consensus assembly using RACA and DESCHRAMBLER increased the N50 by 2.6–53 times compared with those of the initial *de novo* draft assemblies. In addition, the N50 was about 76% of that of the PacBio assembly. Although our IMAP assembly does not show as large N50 as the PacBio assembly, the chromosome-level assembly outcome that was itself obtained by IMAP, which was generated without additional sequencing to improve the initial draft assembly, can contribute significantly to the community. While an assembly generated using the Illumina sequencing data needs several months of extra labor cost and time to finish an assembly [7], IMAP can shorten this step substantially to only several additional hours that are necessary for running the pipeline. Since IMAP uses only Illumina short read sequencing data to build the chromosome-level assembly, this avoids the considerable cost and material requirements that PacBio needs [7, 8].

**Table 1:** Assembly evaluation metrics and results. The W303 strain assembly in each step of IMAP, including the final one, was validated using evaluation metrics such as N50, the length of the shortest contig (MIN), the length of the longest contig (MAX), and total length. We also calculated the fraction of mapped reads and the portion of read pairs that were properly mapped.

Dataset (W303)		MIN (bp)	MAX (bp)	N50 (bp)	Total length (bp)	Mapped reads (%)	Proper pairs (%)
*De novo* assembly	SPAdes	80	515,973	187,035	13,901,101	99.40	96.24
	MaSurCa	300	784,921	273,283	11,838,299	82.86	97.88
	SOAPdenovo2	200	61,911	13,286	11,749,637	50.60	88.48
RACA assembly	RACA-SPAdes	80	1,058,428	716,084	13,905,771	99.40	96.24
	RACA-MaSurCa	300	1,436,612	706,991	11,842,202	82.86	97.88
	RACA-SOAPdenovo2	200	1,076,849	69,631	11,772,637	50.60	88.49
Meta assembly	Meta	80	1,448,740	702,641	13,773,679	98.56	96.19
Final assembly	Corrected assembly	80	1,450,556	705,629	13,847,490	98.57	97.10
PacBio	PacBio	3,688	1,575,129	929,095	12,433,409	99.15	98.73

We also computed the fraction of mapped reads and the portion of proper read pairs out of the mapped reads. The total length of the assembly and the fraction of mapped reads after the DESCHRAMBLER meta-assembly step slightly decreased, compared to the original SPAdes assembly that initially showed the highest value initially. This occurs because some bases in a RACA assembly may be inconsistent with the majority of other RACA assemblies, and in the meta-assembly step they may be discarded. However, the total length and the fraction of mapped reads were quite close to the initial assembly, and the fraction of the proper read pairs in the final assembly was slightly improved, compared to the initial input.

We also applied IMAP for other commonly used yeast strains: SK1 (at approximately 530× coverage; S2 Table) and Sigma1278b (at approximately 191× coverage; S3 Table). For SK1, we also evaluated our IMAP output against a recently available PacBio assembly. For Sigma1278b, our improved assembly could serve as a reference sequence for the strain genome, until any long-read sequencing data are generated. Our assembly of Sigma1278b used the same sequencing data that was built in the original study [22]. Therefore, we could observe a drastic increase in the N50 from 109,268 bp [22] to 719,378 bp in our IMAP results.

We also examined the impact of the choice of a reference genome in our pipeline. We used the SK1 PacBio assembly as another reference for RACA instead of S288C strain genome for W303 sequencing data using IMAP, and the W303 PacBio assembly for SK1. The statistics of these IMAP assembly results for W303 and SK1 (S4 and S5 Tables) were quite similar to the original results (Table 1 and S2 Table) with the S288C strain sequence for the RACA reference. This shows that the assembly output is not much influenced by the choice of the RACA reference as long as it is close enough to the target genome.

**Fig 2:**
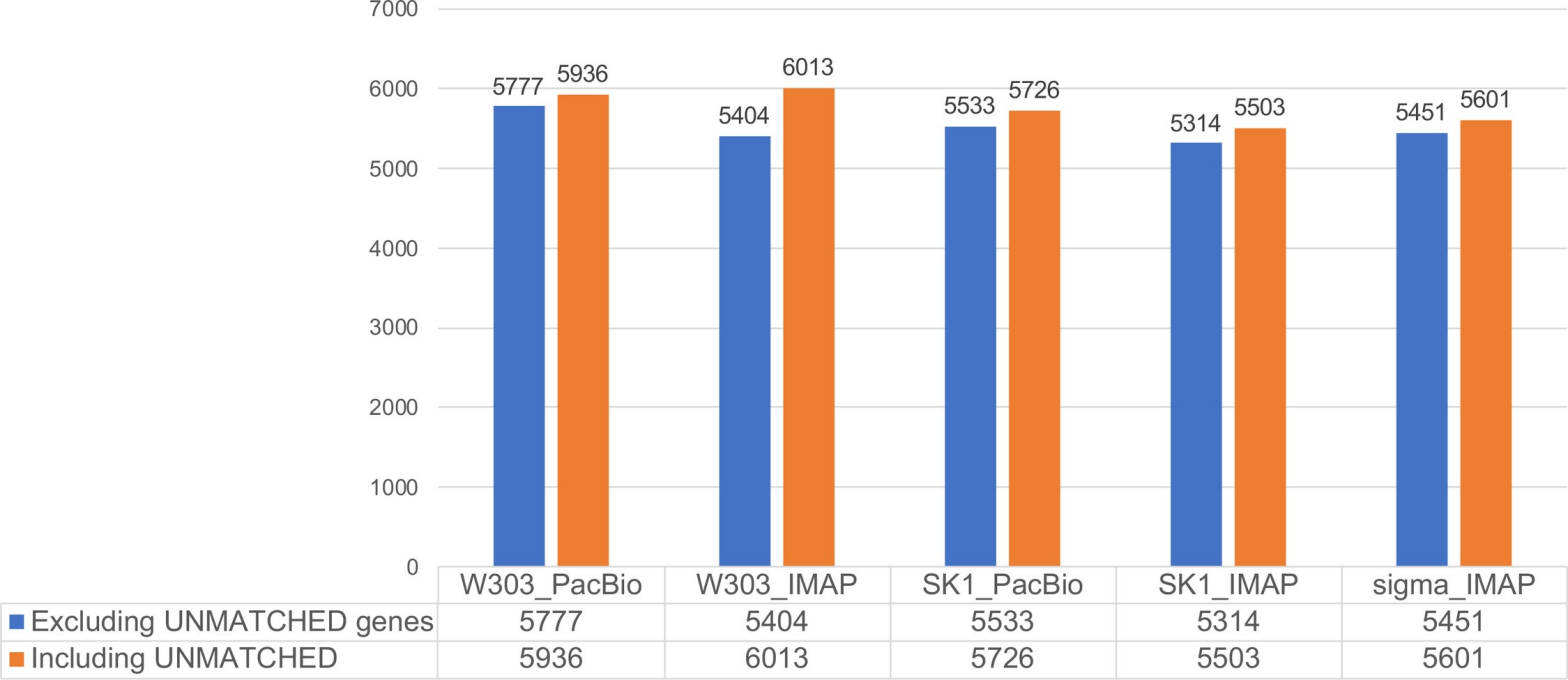
Assembly evaluation using gene annotation. We validated the quality of our assemblies by measuring annotation completeness using AGAPE gene annotation [22]. AGAPE determines genes that are longer than 300 bases, and matches each gene to the NCBI database. If there are no genes found in the NCBI database or genes are partially matched less than a threshold value [22], they are marked as UNMATCHED, and parts of these genes are likely to be missed, because of assembly or annotation errors (note that although UNMATCHED genes could include novel genes, most are partially assembled genes in the *S*. *cerevisiae* strain genome [22]). We evaluated the annotation completeness for the assemblies of the genomes of three yeast strains: W303, SK1, and Sigma1278b. For W303 and SK1, we compared the gene annotation results of our IMAP assembly with that of the PacBio assembly using AGAPE.

We validated the accuracy of the assemblies by measuring annotation completeness using AGAPE gene annotation (Fig 2). We annotated genes with both our IMAP assemblies and PacBio assemblies for the yeast W303, and SK1 strains using AGAPE, and compared their annotation results. AGAPE detects genes longer than 300 bases in a sequence, and searches for their gene names in the NCBI database [22]. If a gene detected by AGAPE is not found in the NCBI database, it is marked as “UNMATCHED”, and part of the gene is likely to be missed because of assembly and annotation errors. We counted the number of genes, both including and excluding the UNMATCHED genes. When the UNMATCHED genes were excluded, our IMAP assemblies for W303 and SK1 had 93.5 and 96%, respectively, of the genes to be determined, relative to the genes annotated using the PacBio assemblies. The initial *de novo* draft assemblies missed scaffolds that contained paralogous genes (480 yeast genes in 197 paralogous groups); therefore, some of them were missed in our IMAP assembly in the AGAPE annotation: 233 fewer paralogous genes for W303, and 177 fewer for SK1 were detected in the IMAP assemblies than in the PacBio assemblies. In most cases, this explained the difference in the number of annotated genes between the IMAP and PacBio assemblies (note that repetitive genomic segments that include similar gene copies are known to be challenging for *de novo* assembly using short-read sequencing only).

For Sigma1278b, we used the same sequencing data as used by the original study [22]. We compared our annotation results with those of the annotation performed in the original study [22]. Our IMAP assembly had 5,451 genes annotated, while the original study [22] reported 5,224 genes, excluding the UNMATCHED genes. This illustrates the improvement in assembly quality gained using IMAP.

When we compared the number of annotated genes among the three *S*. *cerevisiae* strains, we expected similar numbers; however, there was some variation. This might have been caused by the variation in the sequencing depth and quality of the original sequencing data. We also applied IMAP for three commonly used fungal strains: *Aspergillus nidulans* A713, *Neurospora crassa* 73, and *Thielavia terrestris* CBS 492.74. S6−S8 Tables summarize the statistics of their assemblies.

### Synteny mapping of W303 and S288C

**Fig 3:**
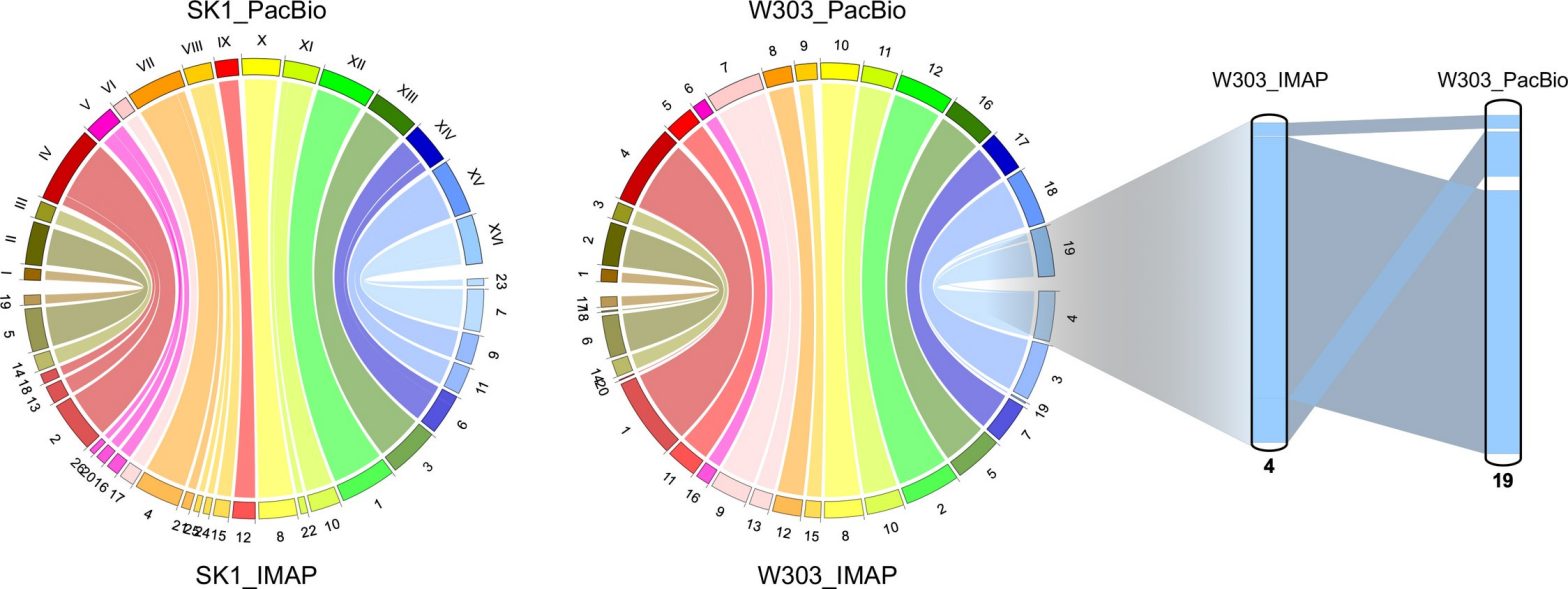
Synteny mapping of the IMAP and PacBio assemblies for strains SK1 and W303. Each color indicates a pair of genomic segments aligned together between the two genomes. Each scaffold at the chromosome level of the IMAP assembly is almost exactly mapped to a chromosome sequence in the PacBio assembly, except for a couple of synteny blocks in W303, as shown in the right panel. In general, there is no significant large-scale structural difference between the IMAP and PacBio assemblies.

We mapped large-scale conserved segments, called synteny blocks, between the IMAP and PacBio assemblies for the SK1 and W303 strain genomes, using LASTZ and software tools available in mySyntenyPortal [32]. Fig 3 shows that each chromosome in our IMAP result is almost exactly mapped to one in the PacBio assembly, excluding two synteny blocks in W303. The additional panel at the right side of Fig 3 shows the synteny blocks in a different order. The discrepancy in the synteny blocks between the IMAP and PacBio assemblies for W303 requires further investigation. We also calculated the percent identity of the alignment between the IMAP and PacBio assemblies generated using LASTZ, and found 100% identity.

In the comparison of the W303 IMAP assembly and the S288C reference sequence using mySyntenyPortal, synteny blocks between the two assemblies were consistent in terms of their order. This agreed with a previous study for the W303 strain genome [34]. In general, there was no significant large-scale difference in terms of the order of synteny blocks between the IMAP and PacBio assemblies.

### Correlation of sequencing depth and assembly quality

The W303 sequence datasets had very deep fold coverage (4,000×); therefore, we assessed the effects of sequencing fold coverage on assembly quality. We sampled sequence datasets randomly to test the assembly for 10×, 20×, 50×, 100×, 200×, 500×, 1,000×, and 4,000× coverage, respectively. The initial datasets were composed of 40 population samples, each of which had 100× coverage. Merging datasets for several samples generated data with multiples of 100× coverage. For example, merging two samples produced sequence data with 200× coverage, while merging five samples produced 500× coverage. To generate the 10×, 20×, and 50× coverage data, we randomly sampled reads from the 4,000× coverage data.

**Fig 4:**
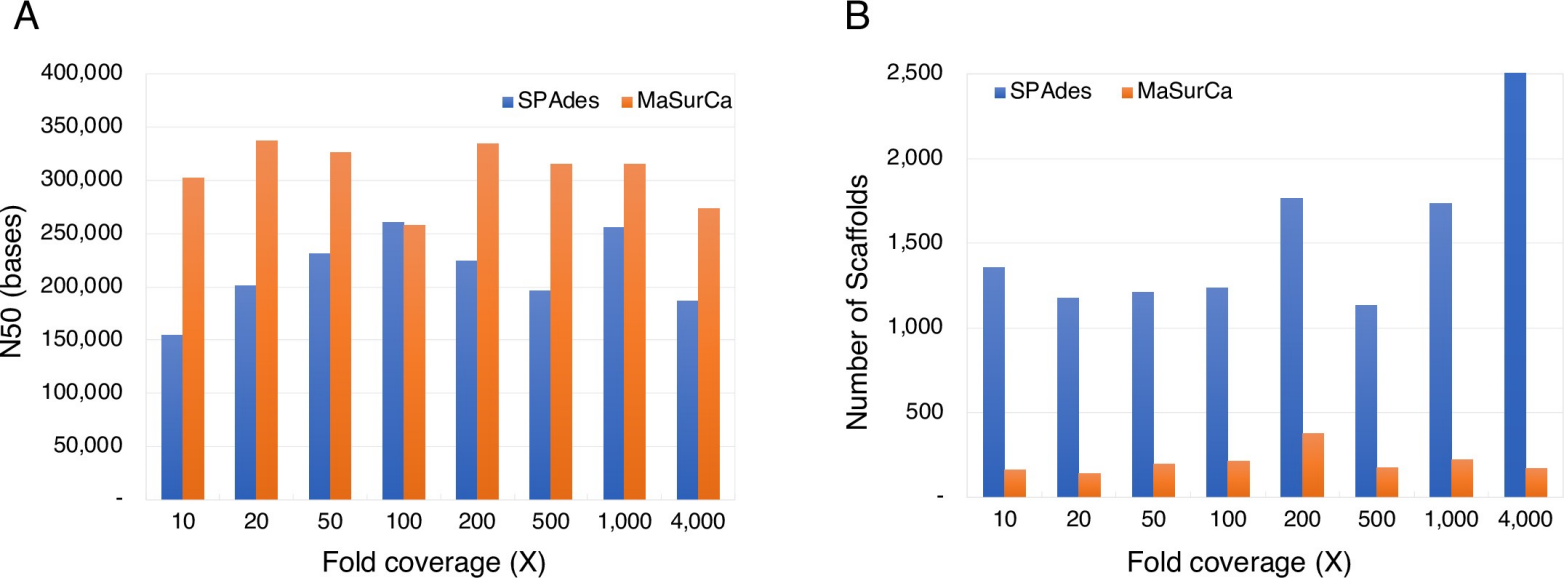
The effects of sequencing fold coverage on assembly quality. We generated sequence datasets with various sequencing fold coverages via random sampling of very deep coverage sequencing data, and applied two assembly pipelines: SPAdes and MaSurCA. (A) The assembly using SPAdes with 100× coverage showed the highest N50 value, while the one using MaSurCA with 20× coverage had the highest N50. (B) Sequencing data at 500× coverage yielded an assembly with the fewest scaffolds using SPAdes, while that with 20× coverage generated the fewest using MaSurCA.

For each dataset, we applied two *de novo* assembly pipelines: SPAdes and MaSurCA. The N50 of the SPAdes assembly was highest at 100× coverage, while that of MaSurCA was highest at 20× coverage (Fig 4A; S9 Table). In terms of the number of scaffolds, SPAdes showed the fewest scaffolds at 500× coverage, while MaSurCA showed the fewest at 20× coverage (Fig 4B; S9 Table). In principle, deeper coverage should generate better quality assemblies. However, we found no correlation between assembly quality and sequencing coverage above 10× coverage. This analysis suggests that 10× coverage may be sufficient enough to obtain a high-quality sequence assembly of yeast strains from short-read sequencing data, and that there is high variability in assembly quality, depending on the initial *de novo* assemblers.

### Description of the usage of IMAP and influence of parameters

Users can install the IMAP package including all prerequisite programs using a Docker image. Docker can be installed by following the instruction in https://www.docker.com/. Once Docker is ready, download the source of IMAP using the command “git clone https://github.com/jkimlab/IMAP.git”, build a Docker image at the directory where “Dockerfile” is located via command “docker build -t [image_name]”, and run the image and create a container using command “docker run -it [image_name] /bin/bash”. Then users can obtain an assembly result using the IMAP Perl script. To run the IMAP Perl script, a parameter file needs to be provided, and the file format and details are available in https://github.com/jkimlab/IMAP.

**Fig 5:**
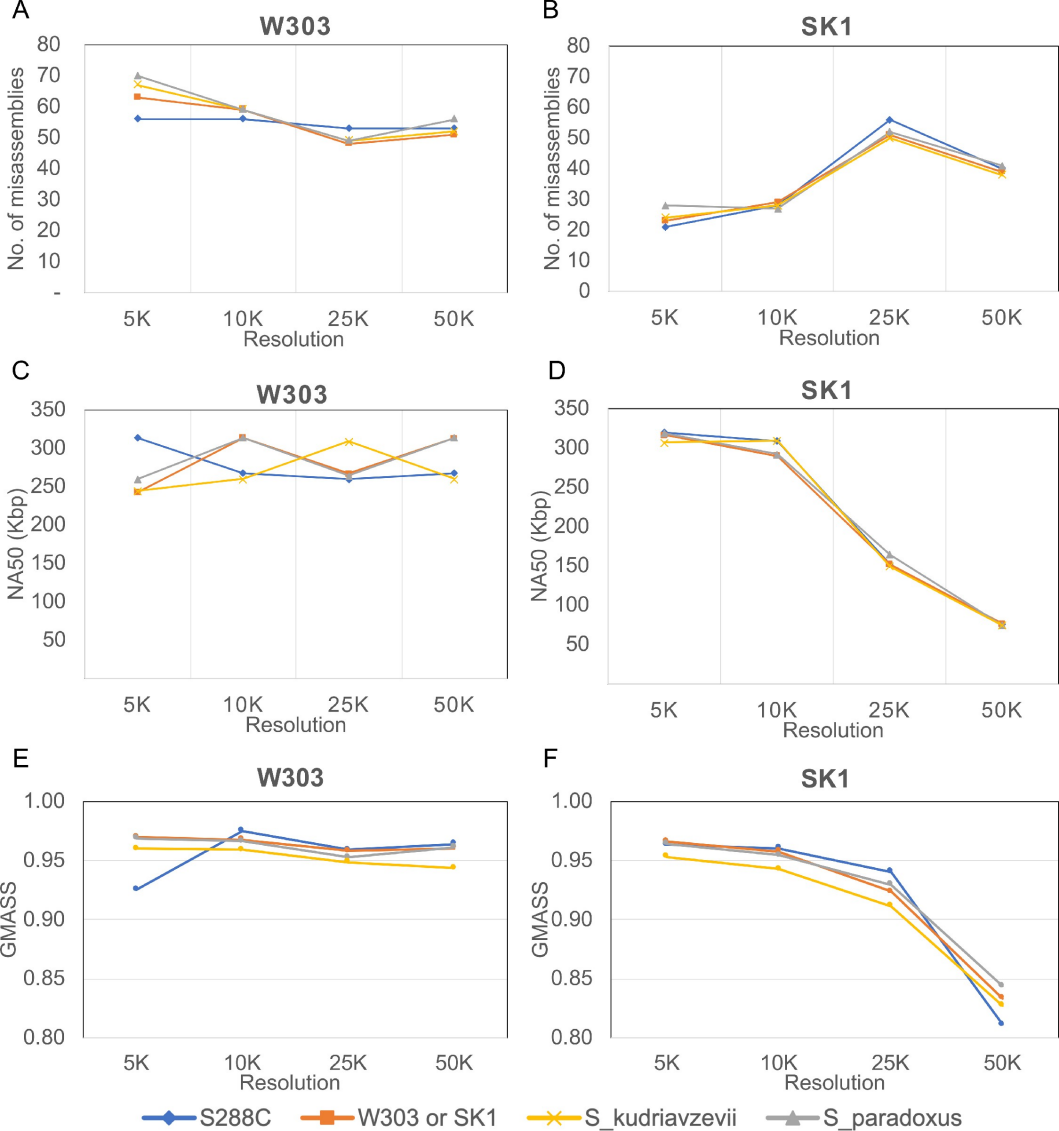
The effects of IMAP parameters on the quality of meta-assemblies. Meta-assemblies of yeast strain W303 and SK1 were generated using different settings of two IMAP parameters (reference species and resolution), and compared with their corresponding PacBio assembly in terms of the number of misassemblies (A and B), NA50 (C and D), and assembly structural similarity (E and F). In this comparison, four different references (S288C, SK1, *S*. *paradoxus*, *S*. *kudriavzevii* for the W303 meta-assembly, and S288C, W303, *S*. *paradoxus*, *S*. *kudriavzevii* for the SK1 meta-assembly), and four different resolutions (5, 10, 25, and 50 Kbp) were used.

There are some major parameters that need to be determined by users such as resolution, which is the minimum length of similar assembly regions (set as 10 Kbp by default), and the genome sequences of a reference species. We examined how these parameters affect the resulting meta-assembly (Fig 5). To see the influence of theses parameters, we built the yeast strain W303 and SK1 assemblies using IMAP with different parameter settings: four different resolutions (5 Kbp, 10 Kbp, 25 Kbp, and 50 Kbp), and four different reference genomes (*S*. *cerevisiae* strain S288C, *S*. *cerevisiae* strain SK1, *S*. *paradoxus*, and *S*. *kudriavzevii* for the W303 assembly, and *S*. *cerevisiae* strain S288C, *S*. *cerevisiae* strain W303, *S*. *paradoxus*, and *S*. *kudriavzevii* for the SK1 assembly). *S*. *cerevisiae* strain S288C is closest and *S*. *kudriavzevii* furthest as the reference for W303 and SK1 [35]. The chromosome-level assemblies of those reference genomes were downloaded from the NCBI database (Accession number GCA_000146045.2 for S288C, GCA_002163515.1 for W303, GCA_002057885.1 for SK1, GCA_002079055.1 for *S*. *paradoxus*, and GCA_003327635.1 for *S*. *kudriavzevii*.

We compared our various assembly results with the W303 and SK1 PacBio assemblies using the assembly assessment tools QUAST [36] and GMASS [37]. We measured the number of misassembled contigs (Fig 5A and 5B; the number of contigs that contain misassembly events relative to the PacBio assembly) and NA50 (Fig 5C and 5D; N50 after fixing misassemblies) using QUAST, and a structural similarity score (Fig 5E and 5F; structural similarity in terms of synteny mapping of our meta-assembly against the PacBio assembly) using GMASS. In terms of the number of misassembled contigs, the reference SK1 with 25 Kbp resolution showed the lowest number for the W303 meta-assembly, and the reference S288C with 5 Kbp resolution for SK1. For NA50, references S288C with 5 Kbp resolution and SK1 with 10 Kbp resolution produced results with the highest value for the W303 meta-assembly, and references S288C and W303 with 5 Kbp resolution for SK1. In the case of the GMASS score, reference S288C with 10 Kbp resolution generated the most similar synteny structures to the PacBio assembly for the W303 meta-assembly, and reference W303 with 5 Kbp resolution for the SK1 result.

These results suggest that using closer reference sequences with 5 or 10 Kbp resolution produces assemblies in high quality, but references at further distances can still generate assemblies in reasonable quality. In addition, the resolution affects the quality of meta-assembly results a little more than the reference setting. The default value (10 Kbp) seems reasonable, but we suggest that users build assemblies using a few different resolution settings.

## Conclusion

We developed a new pipeline, called IMAP, to integrate various *de novo* assembly tools and post-scaffolding steps to extend and order scaffolds to build a chromosome-level genome assembly. We rigorously validated various combinations of approaches in each step and identified the optimal combination of all steps to build the chromosome-level assembly using only short-read sequencing data. We demonstrated the utility of our pipeline by constructing chromosome level assemblies for *S*. *cerevisiae* non-reference strains: W303, SK1, and Sigma1278b, which are the commonly used laboratory strains in yeast genetics studies, and for other fungal genomes, which serve as model organisms in cell biology and genetics studies. There are missing bases in our assemblies of repetitive genomic segments, such as those in Ty elements and telomeric regions, which are known to be very challenging for sequencing assembly. However, the scaffolds of the initial *de novo* draft assemblies were combined and ordered accurately.

In general, much time and effort are required for manual curation and additional computation to complete a reference sequence for a target genome, and to confirm that that sequence could serve as the reference for the community via a consortium. Our pipeline can reduce the time spent on the manual tasks required for curation and discussion. We believe that IMAP could serve as a useful pipeline for collaboration between genomics and bio-curation communities to build reference genome sequences for various organisms. Our study could also help to identify novel evolutionary patterns using chromosome-level assemblies at various temporal or spatial points, using short-read sequencing data.

Long-read sequencing technologies, such as PacBio and Nanopore, are becoming more available, and high-quality assemblies can be obtained with the integration of long- and short-read data. However, for some projects, these new methods are still too expensive, such as for short-term evolution studies using sequencing data, and because of budget limitations that dictate genomics analyses using short-read sequencing data only. IMAP will be helpful in these cases. Assemblies from long-read sequencing can also be easily plugged into our pipeline to improve the assembly quality, by combining long-read results with short-read data. In the future, we will continue to improve our pipeline to build chromosome-level assemblies for larger genomes, such as humans and cancer samples. Our pipeline can accelerate the determination of temporal evolutionary dynamics in cancer genomes at fine-scale resolution, using comparative analyses using sequences assembled at the chromosome level.

## Supporting information

S1 Table Information of data for three fungal strains. (DOCX)

S2 Table Evaluation results for the SK1 strain assembly. (DOCX)

S3 Table Evaluation results for the Sigma 1278b strain assembly. (DOCX)

S4 Table Evaluation results for the W303 strain assembly with reference SK1 PacBio instead of S288C. (DOCX)

S5 Table Evaluation results for the SK1 strain assembly with reference W303 PacBio instead of S288C. (DOCX)

S6 Table Statistics of the assembly of the fungal strain *Aspergillus nidulans* A713. (DOCX)

S7 Table Statistics of the assembly of the fungal strain *Neurospora crassa* 73. (DOCX)

S8 Table Statistics of the assembly of the fungal strain *Thielavia terrestris* CBS 492.74. (DOCX)

S9 Table Statistics of the assemblies of the yeast strain W303 generated using sequencing data with various coverages. (DOCX)
