# Supplementary material for: Integrative Meta-Assembly Pipeline (IMAP): Chromosome-level genome assembler combining multiple de novo assemblies
Source: PLoS One. 2019 Aug 27;14(8):e0221858. doi: 10.1371/journal.pone.0221858 (PMC6711525; doi:10.1371/journal.pone.0221858)
Supplement: S1 Table — (DOCX) [file pone.0221858.s001.docx]

| Species | *Aspergillus nidulans* |
| --- | --- |
| Target strain  (NCBI accession number) | A713 (SRR5749659) |
| Reference strain | FGSC A4 |
| Outgroup | *Aspergillus fumigatus* |
| Source for the reference and outgroup sequences | http://www.aspergillusgenome.org |
|  |  |
| Species | *Neurospora crassa* |
| Target strain  (NCBI accession number) | Strain 73 (SRX872419) |
| Reference strain | NC12 |
| Outgroup | *Neurospora tetrasperma* |
| Source for the reference and outgroup sequences | ftp://ftp.ensemblgenomes.org/pub/fungi |
|  |  |
| Species | *Thielavia terrestris* |
| Target strain  (NCBI accession number) | CBS 492.74 (SRX298659) |
| Reference strain | NRRL 8126 |
| Outgroup | *Thermothelomyces thermophila* ATCC 42464 |
| Source for the reference and outgroup sequences | ftp://ftp.ensemblgenomes.org/pub/fungi |
