# Supplementary material for: Integrative Meta-Assembly Pipeline (IMAP): Chromosome-level genome assembler combining multiple de novo assemblies
Source: PLoS One. 2019 Aug 27;14(8):e0221858. doi: 10.1371/journal.pone.0221858 (PMC6711525; doi:10.1371/journal.pone.0221858)
Supplement: S2 Table — (DOCX) [file pone.0221858.s002.docx]

| Dataset (SK1 with reference S288C) | | MIN  (bp) | MAX  (bp) | N50  (bp) | Total length  (bp) | Mapped reads | Proper pairs |
| --- | --- | --- | --- | --- | --- | --- | --- |
| *De novo* assembly | Spades | 80 | 326,738 | 64,602 | 11,769,237 | 99.67% | 98.02% |
|  | MaSurCa | 301 | 217,864 | 41,266 | 11,111,148 | 75.29% | 97.44% |
|  | SOAPdenovo2 | 100 | 145,188 | 31,924 | 12,788,634 | 99.23% | 94.02% |
| RACA assembly | On Spades | 80 | 972,680 | 482,484 | 11,785,666 | 99.67% | 98.01% |
|  | On MaSurCa | 301 | 913,552 | 602,592 | 11,131,068 | 75.29% | 97.43% |
|  | On SOAPdenovo2 | 100 | 856,147 | 383,323 | 12,810,218 | 99.23% | 94.04% |
| Meta assembly | Meta | 80 | 972,398 | 665,741 | 11,758,028 | 99.46% | 97.98% |
| Final assembly | Corrected-assembly | 80 | 972,610 | 666,314 | 11,760,467 | 99.46% | 98.06% |
| PacBio | PacBio | 84,638 | 1,486,921 | 923,535 | 12,147,923 | 81.48% | 98.93% |
