# Supplementary material for: Integrative Meta-Assembly Pipeline (IMAP): Chromosome-level genome assembler combining multiple de novo assemblies
Source: PLoS One. 2019 Aug 27;14(8):e0221858. doi: 10.1371/journal.pone.0221858 (PMC6711525; doi:10.1371/journal.pone.0221858)
Supplement: S3 Table — (DOCX) [file pone.0221858.s003.docx]

| Dataset (Sigma1278b with reference S288C) | | MIN  (bp) | MAX  (bp) | N50  (bp) | Total length  (bp) | Mapped reads | Proper pairs |
| --- | --- | --- | --- | --- | --- | --- | --- |
| *De novo* assembly | Spades | 80 | 454,811 | 138,590 | 11,694,367 | 99.84% | 96.65% |
|  | MaSurCa | 160 | 442,566 | 97,733 | 12,367,860 | 97.37% | 96.36% |
|  | SOAPdenovo2 | 100 | 667,982 | 175,806 | 11,845,136 | 99.45% | 90.16% |
| RACA assembly | On Spades | 80 | 1,229,127 | 717,070 | 11,702,200 | 99.84% | 96.68% |
|  | On MaSurCa | 160 | 1,437,493 | 659,302 | 12,380,820 | 97.38% | 96.45% |
|  | On SOAPdenovo2 | 100 | 1,430,785 | 717,133 | 11,850,891 | 99.45% | 90.17% |
| Meta assembly | Meta | 80 | 1,435,360 | 716,705 | 11,691,584 | 99.81% | 96.64% |
| Final assembly | Corrected-assembly | 80 | 1,440,411 | 719,378 | 11,716,077 | 99.81% | 96.93% |
| PacBio | PacBio | 80 | 454,811 | 138,590 | 11,694,367 | 99.84% | 96.65% |
