# Supplementary material for: Integrative Meta-Assembly Pipeline (IMAP): Chromosome-level genome assembler combining multiple de novo assemblies
Source: PLoS One. 2019 Aug 27;14(8):e0221858. doi: 10.1371/journal.pone.0221858 (PMC6711525; doi:10.1371/journal.pone.0221858)
Supplement: S4 Table — (DOCX) [file pone.0221858.s004.docx]

| Dataset (W303 with reference SK1 PacBio) | | MIN  (bp) | MAX  (bp) | N50  (bp) | Total length  (bp) | Mapped reads | Proper pairs |
| --- | --- | --- | --- | --- | --- | --- | --- |
| *De novo* assembly | Spades | 80 | 515,973 | 187,035 | 13,901,101 | 98.52% | 93.05% |
|  | MaSurCa | 300 | 784,921 | 273,283 | 11,838,299 | 80.92% | 95.12% |
|  | SOAPdenovo2 | 200 | 61,911 | 13,286 | 11,749,637 | 40.87% | 85.05% |
| RACA assembly | On Spades | 80 | 1,450,585 | 777,307 | 13,905,763 | 98.52% | 93.06% |
|  | On MaSurCa | 300 | 1,401,523 | 730,449 | 11,842,302 | 80.92% | 95.12% |
|  | On SOAPdenovo2 | 200 | 816,198 | 44,423 | 11,773,037 | 40.88% | 85.05% |
| Meta assembly | Meta | 80 | 908,987 | 273,486 | 13,728,209 | 97.40% | 92.99% |
| Final assembly | Corrected-assembly | 80 | 922,110 | 273,557 | 13,787,123 | 97.43% | 93.82% |
| PacBio | PacBio | 3,688 | 1,575,129 | 929,095 | 12,433,409 | 98.39% | 95.35% |
