# Supplementary material for: Integrative Meta-Assembly Pipeline (IMAP): Chromosome-level genome assembler combining multiple de novo assemblies
Source: PLoS One. 2019 Aug 27;14(8):e0221858. doi: 10.1371/journal.pone.0221858 (PMC6711525; doi:10.1371/journal.pone.0221858)
Supplement: S5 Table — (DOCX) [file pone.0221858.s005.docx]

| Dataset (SK1 with reference W303 PacBio) | | MIN  (bp) | MAX  (bp) | N50  (bp) | Total length  (bp) | Mapped reads | Proper pairs |
| --- | --- | --- | --- | --- | --- | --- | --- |
| De novo assembly | Spades | 80 | 326,738 | 64,602 | 11,769,237 | 99.37% | 98.27% |
|  | MaSurCa | 301 | 217,864 | 41,266 | 11,111,148 | 73.40% | 98.77% |
|  | SOAPdenovo2 | 100 | 145,188 | 31,924 | 12,788,634 | 98.09% | 94.17% |
| RACA assembly | On Spades | 80 | 886,626 | 480,147 | 11,785,518 | 99.37% | 98.28% |
|  | On MaSurCa | 301 | 1,145,201 | 701,094 | 11,130,338 | 73.41% | 98.78% |
|  | On SOAPdenovo2 | 100 | 811,714 | 503,592 | 12,809,357 | 98.10% | 94.20% |
| Meta assembly | Meta | 80 | 665,560 | 321,811 | 11,753,965 | 99.17% | 98.26% |
| Final assembly | Corrected-assembly | 80 | 665,999 | 321,830 | 11,755,999 | 99.17% | 98.33% |
| PacBio | PacBio | 84,638 | 1,486,921 | 923,535 | 12,147,923 | 81.00% | 99.44% |
