# Supplementary material for: Integrative Meta-Assembly Pipeline (IMAP): Chromosome-level genome assembler combining multiple de novo assemblies
Source: PLoS One. 2019 Aug 27;14(8):e0221858. doi: 10.1371/journal.pone.0221858 (PMC6711525; doi:10.1371/journal.pone.0221858)
Supplement: S6 Table — (DOCX) [file pone.0221858.s006.docx]

| Dataset (*Aspergillus nidulans* A713) | | No. of scaffolds | MIN  (bp) | MAX  (bp) | N50  (bp) | Total length  (bp) |
| --- | --- | --- | --- | --- | --- | --- |
| *De novo* assembly | Spades | 742 | 80 | 1,200,208 | 311,218 | 30,011,837 |
|  | MaSurCa | 340 | 334 | 1,134,138 | 353,813 | 30,479,586 |
|  | SOAPdenovo2 | 1,444 | 100 | 1,227,751 | 461,367 | 30,157,537 |
| RACA assembly | On Spades | 603 | 80 | 5,026,125 | 3,284,219 | 30,022,609 |
|  | On MaSurCa | 219 | 334 | 6,604,879 | 4,850,733 | 30,490,986 |
|  | On SOAPdenovo2 | 1,349 | 100 | 4,841,849 | 3,697,568 | 30,165,707 |
| Meta assembly | Meta | 601 | 80 | 4,848,164 | 3,679,644 | 29,994,799 |
| Final assembly | Corrected-assembly | 601 | 80 | 4,856,531 | 3,691,639 | 30,066,383 |
