# Supplementary material for: Integrative Meta-Assembly Pipeline (IMAP): Chromosome-level genome assembler combining multiple de novo assemblies
Source: PLoS One. 2019 Aug 27;14(8):e0221858. doi: 10.1371/journal.pone.0221858 (PMC6711525; doi:10.1371/journal.pone.0221858)
Supplement: S7 Table — (DOCX) [file pone.0221858.s007.docx]

| Dataset (*Neurospora crassa* 73) | | No. of scaffolds | MIN  (bp) | MAX  (bp) | N50  (bp) | Total length  (bp) |
| --- | --- | --- | --- | --- | --- | --- |
| *De novo* assembly | Spades | 605 | 80 | 683,698 | 192,428 | 41,272,095 |
|  | MaSurCa | 756 | 115 | 854,345 | 261,629 | 41,732,769 |
|  | SOAPdenovo2 | 1,703 | 100 | 932,517 | 237,836 | 41,393,477 |
| RACA assembly | On Spades | 301 | 80 | 10,145,443 | 6,312,989 | 41,289,943 |
|  | On MaSurCa | 515 | 115 | 9,894,609 | 6,062,679 | 41,751,883 |
|  | On SOAPdenovo2 | 1,429 | 100 | 9,692,977 | 6,071,702 | 41,414,828 |
| Meta assembly | Meta | 301 | 80 | 9,713,446 | 6,066,864 | 41,121,872 |
| Final assembly | Corrected-assembly | 301 | 80 | 9,716,826 | 6,067,908 | 41,139,400 |
