# Supplementary material for: Integrative Meta-Assembly Pipeline (IMAP): Chromosome-level genome assembler combining multiple de novo assemblies
Source: PLoS One. 2019 Aug 27;14(8):e0221858. doi: 10.1371/journal.pone.0221858 (PMC6711525; doi:10.1371/journal.pone.0221858)
Supplement: S8 Table — (DOCX) [file pone.0221858.s008.docx]

| Dataset (*Thielavia terrestris* CBS 492.74) | | No. of scaffolds | MIN  (bp) | MAX  (bp) | N50  (bp) | Total length  (bp) |
| --- | --- | --- | --- | --- | --- | --- |
| *De novo* assembly | Spades | 831 | 80 | 602,123 | 136,093 | 36,932,491 |
|  | MaSurCa | 280 | 300 | 3,274,653 | 976,165 | 37,049,951 |
|  | SOAPdenovo2 | 1,880 | 100 | 3,112,864 | 529,459 | 37,195,289 |
| RACA assembly | On Spades | 471 | 80 | 8,791,094 | 4,714,596 | 36,955,485 |
|  | On MaSurCa | 228 | 300 | 9,807,924 | 4,749,043 | 37,054,097 |
|  | On SOAPdenovo2 | 1,785 | 100 | 10,314,398 | 5,489,663 | 37,203,385 |
| Meta assembly | Meta | 474 | 80 | 8,185,822 | 4,247,520 | 36,192,481 |
| Final assembly | Corrected-assembly | 474 | 80 | 8,230,513 | 4,260,851 | 36,347,827 |
