# Supplementary material for: Integrative Meta-Assembly Pipeline (IMAP): Chromosome-level genome assembler combining multiple de novo assemblies
Source: PLoS One. 2019 Aug 27;14(8):e0221858. doi: 10.1371/journal.pone.0221858 (PMC6711525; doi:10.1371/journal.pone.0221858)
Supplement: S9 Table — (DOCX) [file pone.0221858.s009.docx]

| Assembler (coverage) | Total length (bp) | No. of scaffolds | N50  (bp) | Min  (bp) | Max  (bp) |
| --- | --- | --- | --- | --- | --- |
| spades (10x) | 11,758,071 | 1,357 | 154,803 | 80 | 473,367 |
| spades (20x) | 11,785,577 | 1,175 | 200,813 | 80 | 744,478 |
| spades (50x) | 11,759,203 | 1,209 | 230,878 | 80 | 744,081 |
| spades (100x) | 11,750,388 | 1,235 | 260,492 | 80 | 811,960 |
| spades (200x) | 11,926,916 | 1,766 | 224,671 | 80 | 516,153 |
| spades (500x) | 11,756,745 | 1,135 | 196,398 | 80 | 516,070 |
| spades (1000x) | 11,924,513 | 1,735 | 256,116 | 80 | 742,858 |
| spades (4000x) | 13,901,101 | 5,848 | 187,035 | 80 | 515,973 |
| masurca (10x) | 11,739,366 | 161 | 302,148 | 276 | 1,232,563 |
| masurca (20x) | 11,664,165 | 141 | 337,020 | 277 | 737,888 |
| masurca (50x) | 11,949,637 | 195 | 326,519 | 261 | 740,950 |
| masurca (100x) | 12,230,369 | 214 | 257,634 | 249 | 1,073,139 |
| masurca (200x) | 12,039,019 | 378 | 334,256 | 300 | 990,583 |
| masurca (500x) | 12,026,216 | 177 | 315,380 | 300 | 738,079 |
| masurca (1000x) | 11,927,734 | 221 | 315,261 | 300 | 738,468 |
| masurca (4000x) | 11,838,299 | 170 | 273,283 | 300 | 784,921 |
